# Are all homebound older adults frail?

**DOI:** 10.1093/geroni/igaa057.2812

**Published:** 2020-12-16

**Authors:** Orla Sheehan, Karen Bandeen-Roche, Christine Ritchie, Shang-En Chung, Jeremy Walston, David Roth, Bruce Leff

**Affiliations:** 1 Johns Hopkins University School of Medicine, Baltimore, Maryland, United States; 2 Johns Hopkins Bloomberg School of Public Health, Baltimore, Maryland, United States; 3 UCSF, San Francisco, California, United States; 4 Johns Hopkins University, Baltimore, Maryland, United States

## Abstract

Seven million adults in the United States are homebound and suffer the negative, powerful synergies of multiple chronic conditions, functional impairment, social stressors, and limited social capital. The prevalence of frailty in this vulnerable homebound population is unknown. Using representative data from the National Health and Aging Trends study (NHATS) study linked to Medicare claims (n=4756) we sought to assess the prevalence of frailty in the homebound population (n=361). Among the homebound, 68.5% met the frailty criteria compared to 12.3% of the non-homebound population. The frail homebound had lower educational attainment, were more likely to live alone, self-reported poorer health and more chronic physical and mental health conditions than the non-frail homebound (p<0.05 for all). Frail homebound older adults utilized more health services utilization than non-frail homebound and were twice as likely to be hospitalized (49.8% versus 28.0%, p=0.004).

